# Consequences of lower extremity and trunk muscle fatigue on balance and functional tasks in older people: A systematic literature review

**DOI:** 10.1186/1471-2318-10-56

**Published:** 2010-08-17

**Authors:** Jorunn L Helbostad, Daina L Sturnieks, Jasmine Menant, Kim Delbaere, Stephen R Lord, Mirjam Pijnappels

**Affiliations:** 1 Department of Neuroscience, NTNU, Trondheim, Norway; 2 Department of Geriatrics, St. Olav University Hospital, Trondheim, Norway; 3 Falls and Balance Research Group, Prince of Wales Medical Research Institute, University of New South Wales, Sydney, Australia; 4 Department of Experimental-Clinical and Health Psychology, Faculty of Psychology and Educational Sciences, Ghent University, Belgium; 5 Department of Rehabilitation Sciences and Physiotherapy, Faculty of Medicine and Health Sciences, Ghent University, Belgium; 6 Research Institute MOVE, Faculty of Human Movement Sciences, VU University, Amsterdam, the Netherlands

## Abstract

**Background:**

Muscle fatigue reduces muscle strength and balance control in young people. It is not clear whether fatigue resistance seen in older persons leads to different effects. In order to understand whether muscle fatigue may increase fall risk in older persons, a systematic literature review aimed to summarize knowledge on the effects of lower extremity and trunk muscle fatigue on balance and functional tasks in older people was performed.

**Methods:**

Studies were identified with searches of the PUBMED and SCOPUS data bases.

Papers describing effects of lower extremity or trunk muscle fatigue protocols on balance or functional tasks in older people were included. Studies were compared with regards to study population characteristics, fatigue protocol, and balance and functional task outcomes.

**Results:**

Seven out of 266 studies met the inclusion criteria. Primary findings were: fatigue via resistance exercises to lower limb and trunk muscles induces postural instability during quiet standing; induced hip, knee and ankle muscle fatigue impairs functional reach, reduces the speed and power of sit-to-stand repetitions, and produces less stable and more variable walking patterns; effects of age on degree of fatigue and rate of recovery from fatigue are inconsistent across studies, with these disparities likely due to differences in the fatigue protocols, study populations and outcome measures.

**Conclusion:**

Taken together, the findings suggest that balance and functional task performance are impaired with fatigue. Future studies should assess whether fatigue is related to increased risk of falling and whether exercise interventions may decrease fatigue effects.

## Background

Loss of functional ability and impaired balance control in older people is well documented and attributed to age-related sarcopenia and sensorimotor decline. Another factor, likely to further affect an older person's functional abilities is muscle fatigue. Studies in young people show that muscle fatigue in the lower limbs increases postural sway [1,2], that fatiguing postural back muscles impairs head and trunk control while walking [3], and that quadriceps muscle fatigue alters gait parameters related to slip propensity [4].

Effects of fatigue of specific muscle groups on muscular performance (e.g. strength) in both young and older people has been investigated extensively (see Kent-Braun 2009 [5] for review). Interestingly, despite typical loss in muscle strength with increasing age, several studies have demonstrated that older people are more resistant to muscle fatigue than young people following isometric and dynamic lower extremity muscle work [6-14]. Possible mechanisms for this include lower maximal motor units discharge rates, slower contractile properties and greater reliance on oxidative metabolism in older people [5]. However, not all research supports relative muscle fatigue resistance in older persons [15,16]. In addition, fatigue resistance in older people is not evident for the trunk muscles [17], and one study showed recovery rate after fatigue to be slower in older fallers compared to older non-fallers and young adults [13].

In order to understand whether muscle fatigue in older persons affects fall risk, greater focus should be on consequences for balance and functional performance. Therefore, this systematic literature review assessed the effects of lower extremity and trunk muscle fatigue (seen as a decrement in performance following exercise) and recovery on balance and functional tasks in older people and synthesized the findings with regard to how age, gender, fatigue protocols and effect of fatiguing different muscle groups affect balance and functional abilities. This knowledge may assist in designing falls risk assessments and exercise programs in clinical settings for older people.

## Methods

### Search strategy and selection criteria

We conducted a systematic literature review until June 2009, with a primary search in the PUBMED and a secondary search in the SCOPUS databases. The PUBMED search was performed for the MeSH term "Muscle Fatigue" and the search limited to Human studies, English language, and subsets of Aged 65+ and 80+. The SCOPUS search used the following terms: TITLE(fatigue AND (muscle OR physical)) AND KEY(aging OR ageing OR older OR elder* OR aged) AND (LIMIT-TO(DOCTYPE,"ar")) AND (LIMIT-TO(LANGUAGE,"English")). Additionally, we searched for the terms "fatigue" AND "older" in relevant journals within gerontology and geriatric medicine (Journals of Gerontology and Journal of the American Geriatric Society) and physiology (European Journal of Applied Physiology, Journal of Applied Physiology, Muscle and Nerve and Electromyography and Clinical Neurophysiology). Duplicates between searches were removed.

### Inclusion criteria and quality assessment

To be included, papers had to describe muscle fatigue protocols for the lower extremities or trunk muscles (except diaphragm or pelvic floor muscles). Papers were excluded if they were reviews, methodological or descriptive papers, the focus was on patient groups, all participants were younger than 50 years of age, fatigue was only measured by self-report, or studies were performed in animals or *in vitro*. Finally, papers reporting effect of muscle fatigue on local muscles and not on balance or functional tasks were also excluded.

### Abstraction of data and analysis

Five authors contributed to the review process. Every title and abstract was screened by two randomly allocated reviewers, using the inclusion criteria. A full paper review was performed for the remaining publications by two other randomly allocated reviewers. For the included papers, a first reviewer reported on study aims, population, fatigue protocol, how fatigue was measured, and specification of outcomes and on findings. A second reviewer provided additional details as deemed necessary.

For descriptions of outcomes we divided balance and functional tasks into quiet standing, voluntary movements or external perturbations.

## Results and Discussion

### Search Results

#### Studies identified

The PubMed-search revealed 130 hits and the Scopus-search 170 hits, of which 37 were duplicates. Additionally, three papers were detected through searching selected journals, leaving 266 papers for abstract review. Following abstract review 210 papers were excluded, leaving 56 papers for a full review. Of these, 36 papers reported fatigue of lower extremity or trunk muscles in older people. However, 29 did not have a balance or a functional task outcome, leaving seven papers for final inclusion [18-24].

#### Study populations

Study populations, fatigue protocols and outcome measures are summarized in Table 1. Five studies included a sample of older people in addition to a control group; four young [18-21] and one older [22] control group. Two studies had no control group [23,24]. The sample sizes ranged from 11-24 participants. Mean age of the older people varied from 62.2 to 78.2 years, with a range across studies from 55 to 86 years. One study included only women [23], one only men [20], and five both genders [18,19,21,22,24], of which only Petrella et al. [21] examined gender differences.

**Table 1:** Included studies reported by populations, fatigue protocols and outcome measures

STUDY	POPULATION		FATIGUE PROTOCOL					OUTCOME		
	**Study** **population**	**Control** **group**	**Uni/bilateral** **muscle** **group(s)**	**Intensity**	**Sets &** **rate or** **speed**	**Total** **reps &** **duration**	**Endpoint** **(force/** **velocity** **drop)**	**Functional** **task (pre** **versus post** **fatigue)**	**Main** **fatigue** **effect**	**Fatigue** **×** **Age** **interaction**

Bellew et al. 2006 [23]	18 older adults 77 ± 6 yrs All ♀	-	*Unilateral* Knee flexors/ extensors Ankle plantar/ dorsi-flexors	Peak torque	*Number of* *sets not* *specified* at 180°/s	*Not* *specified*	< 50% peak torque	*Quiet standing * Single-limb stance time *Voluntary* *movements* Modified functional reach Lower extremity reach	Yes Yes Yes	*N/A*

Davidson et el. 2009 [18]	16 older adults 62.2 ± 5.1 yrs 8 ♂, 8 ♀	16 younger adults 9.4 ± 1.4 yrs 8 ♂, 8 ♀	*Not specified:* Ankle plantar flexors *Bilateral:* Lumbar extensors	45% of MVC	1 set of 23 reps/min *Rate not* *specified*	~322 reps (~14 min)	~70% MVC	*External* *perturbations* Maximum forward trunk perturbation withstood without stepping	Yes *	Yes: effect Y < O

Lin et al. 2009 [19]	16 older adults 55-65 yrs ♂ and ♀	16 young adults 18-24 yrs ♂ and ♀	*Unilateral* Ankle plantar- flexors Knee extensors Lumbar extensors	60% of MVC	1 set of 12 reps/min at 60°/s	~144 reps (~12 min)	~60% MVC	*Quiet standing* 11 trials of 75 s at 0.75 - 20 min post fatigue compared to 3 trials pre fatigue	Yes *	Yes: effect Y > O

Mademli et al. 2008 [20]	11 older adults 65 ± 3 yrs All ♂	11 young adults 32 ± 7 yrs All ♂	*Bilateral* Knee extensors/ flexors	25% of MVC	3 sets of 15 reps/min, with 2 min resting intervals at 45°/s	y: ~80 reps; (6.6 ± 3.6 min) o:~100 reps (8.4 ± 5.3 min)	~75-80% MVC	*External* *perturbations* Single step during a forward fall	No *	No: effect Y = O

Moore et al. 2005 [24]	21 older adults 71.2 ± 3.8 yrs, Range 66-81 5 ♂, 16 ♀	-	*Unilateral* Knee flexors/ extensors Ankle plantar/ dorsiflexors Hip abductors/ extensors	60% of 1 RM	3 sets of 10-12 reps, with 2 min resting intervals *Rate not* *specified*	~30 - 36 reps	*Not* *specified*	*Quiet* *standing* 10 trials of 30 s with 10 s resting intervals	Yes	*N/A*

Helbostad et al. 2007 [22]	22 fatigued older adults 78.2 ± 5.0 yrs, Range 70-86 5 ♂, 17 ♀	22 unfatigued older adults 80.4 ± 4.9 yrs, Range 73-89 5 ♂ 17 ♀	*Bilateral* *sit-to-stand* Leg extensors	*Not* *specified*	Fast speed up and down	~ 25 reps	12% mean velocity drop 15% peak velocity drop	*Voluntary* *movements* Walking at habitual gait speed	Yes #	*N/A*

Petrella et al. 2005 [21]	24 older adults 63 ± 0.8 yrs, Range 60-75 13 ♂, 11 ♀	28 young adults 26.9 ± 0.7 yrs, Range 20-35 12 ♂, 16 ♀	*Bilateral* *sit-to-stand* Leg extensors	*Not* *specified*	Fast speed up, slow speed down	10 reps	27% power drop 12% force drop 20% velocity drop	*Voluntary* *movements* Sit-to-stand velocity	Yes *	No: effect Y = O

Y = young; O = older; MVC = Maximum Voluntary Capacity; RM = repetition maximum; N/A = not applicable; Note: * main effect over age groups; # effect in fatigued group only

All studies involved healthy community-dwelling older participants without any musculoskeletal disorders, except for Helbostad et al. [22], who reported prevalence of eleven diseases with 45% of participants having hypertension, 41% incontinence, and 36% stroke or osteoporosis. Helbostad et al. further reported of  41% with a fall the previous year, while two other studies explicitly stated that participants had no history of falls in the past year [18,19]. Participants in the study by Mademli et al. [20] were active in sports, while two studies explicitly reported exclusion of participants engaged in resistance training during the past six months [24] or five years [21].

#### Fatigue protocols and outcome measures

In five studies, the fatigue protocol was performed on one or more specific muscle groups using a dynamometer, at a load related to the participants' maximum voluntary capacity (MVC) [18-20,23,24]. In two studies, lower limb muscle fatigue was performed by sit-to-stand repetitions [21,22]. Participants were instructed to cross arms and repeatedly rise from a chair to an erect position at a fast speed, then sit down either at a fast [22] or slow speed [21]. While Petrella et al. [21] examined fatigue effects over 10 sit-to-stand repetitions, Helbostad et al. [22] required participants to perform sit-to-stand trials until they felt too exhausted to continue.

Four studies fatigued muscle groups over multiple joints [18,19,23,24] with three examining differential effects between the muscle groups [18,19,23]. Five studies sought to fatigue knee-flexors and/or extensors [19-21,23,24], three ankle plantar- and/or dorsi-flexors [18,19,23,24], one hip-abductors and extensors [24], and two fatigued lumbar extensors [18,19]. The two studies employing sit-to-stand repetitions [21,22] did not specify muscle groups that were fatigued.

Three studies fatigued one or more specific muscle group unilaterally in the dominant leg [19,23,24], despite the outcome measure being two-legged standing. Four studies used a bilateral fatigue task to study effects on voluntary movements including sit-to-stand [21], walking [22] and balance recovery from external perturbations [18,20].

For the five studies using isokinetic contraction fatigue protocols [18-20,23,24], force levels varied from 25-60% MVC. Contraction velocity was specified in three studies and ranged from 45-180°/s [19,20,23]. Number of repetitions for the sit-to-stand protocols were 10 [21] and ~25 [22], while other exercises employed between ~30 [24] and ~322 [18] repetitions. Time to perform the fatigue protocol was reported for three studies [18-20] and varied between 8.4 and 14 minutes.

The endpoints of fatigue protocols reported were either: participants unable to lift a weight through the whole range of motion [20,24], performance dipped below a pre-defined threshold over three consecutive repetitions [19,23], participants were apparently exhausted [22], or were based on a pre-defined number of repetitions [21] or time duration [18]. The relative force decline at the endpoint, explicitly reported in two studies [18,23] and assumed in two others [19,20], varied between 50 and 70% of maximum pre-fatigue force level. One study [21] described the effect of fatigue in terms of velocity reduction over repetitions, while another did not specify the fatigue endpoint [24].

Outcome measures varied between studies. Three studies examined quiet stance, during which Lin et al. [19] and Moore et al. [24] calculated centre of pressure (COP) profiles, while Bellew et al. [23] measured single limb stance time. Three studies involved voluntary movements as outcome measures: upper and lower extremity reach length [23], sit-to-stand performance [21] and walking at preferred gait speed [22]. Two studies examined balance recovery from external perturbations in standing: sudden release from a forward leaning position [20] and a perturbation applied to the upper trunk [18].

Only one study examined effects of recovery rate on balance performance [19].

### Results on Fatigue Effects

This systematic literature review examined the effects of lower limb and trunk muscle fatigue on balance and functional tasks in older people. Since none of the manuscripts aimed to study the specific mechanisms of fatigue, fatigue was defined as a decrement in performance following exercise. Six out of seven studies found a significant effect of fatigue on balance in older people [18,19,21-24] while one showed no effect [20]. The studies varied considerably in study populations, fatigue protocols and outcome measures.

#### Main effects of fatigue

Three studies demonstrated that balance performance during quiet stance was poorer after fatigue, as evidenced by reduced single limb stance time [23] and increased postural sway [19,24]. Single limb stance time was reduced following repeated isokinetic knee-flexion/extension and dorsi/plantar-flexion exercises [23]. Similarly, postural sway was increased, evidenced by increased COP displacement while standing following repeated hip-, knee- and ankle-flexion/extension resistance exercises [24]. Finally, a detrimental effect of fatigue on various postural sway parameters was found after fatiguing the ankle plantar-flexors, knee-extensors, lumbar-extensors and shoulder-extensors [19]. The amount, speed and frequency parameters of postural sway are commonly used indicators of postural stability, while single leg stance time is a simple measure of postural stability while balancing on one leg. Taken together, these results suggest that fatigue via resistance exercises to lower limb and trunk muscles generally induces postural instability during quiet standing.

Three studies demonstrated that balance performance during voluntary movements was poorer after fatigue, as evidenced by impaired functional reach [23], reduced speed and power of sit-to-stand repetitions [21] and altered gait parameters [22]. This fatigue-induced increase in step width, medio-lateral trunk accelerations and variability in antero-posterior acceleration is related to decreased walking stability and is comparable to gait patterns in frail people [22]. These studies suggest that induced fatigue of hip, knee and ankle muscles impairs functional reach [23], reduces the speed and power of sit-to-stand repetitions [21] and produces less stable and more variable walking patterns [22].

Contrasting results were found for the effects of fatigue on balance reactions to unexpected perturbations [18,20]. Davidson et al. [18] found balance recovery, following a perturbation to the trunk, was impaired after fatiguing ankle plantar-flexors and lumbar-extensors. Conversely, Mademli et al. [20] showed no effect of knee flexor and extensor fatigue on balance recovery following a sudden release from a supported leaning posture after fatigue exercise at 25% MVC. However, the lack of effect in the latter study might be explained by the fatigue protocol which involved a low relative force or sub-optimal protocol relative to outcome measure (i.e. the knee muscles fatigued but the hip muscles are required for balance recovery) may explain the lack of a fatigue effect in the latter study.

#### Effect of age

Four studies [18-21] examined whether fatigue effects on balance and functional tasks differed between young and older people. No age-related fatigue differences were reported for responses to external perturbations [20] and sit-to-stand performance [21]. Davidson et al. [18] examined 13 different balance response variables (perturbation magnitude withstood and a range of COM and COP measures) following an unexpected perturbation. No age-related differences were found for 10 variables. However, compared to young, older people had significantly increased COP peak velocity, time-to-peak velocity and time-to-return to the original COP position following the perturbation in the fatigued state. These findings suggest that, relative to young, older people's balance was more challenged by the perturbation following fatigue. In contrast to this, Lin et al. [19] found postural sway during quiet standing to be more affected by fatigue in young, compared to older people. These contrasting results could indicate that different strategies may be adopted in young and older people in static and dynamic balance tasks with fatigue (for example, older people might rely more on a hip strategy to control postural sway under fatigued conditions compared to young). Further work is required to test this hypothesis.

Several studies investigating muscle fatigue on muscle strength and function have suggested that older people are more fatigue-resistant than younger people [8-14,25]. Three mechanisms have been proposed. First, the majority of these studies suggest that fatigue resistance in older people is likely due to morphological mechanisms, rather than central or peripheral activation changes. Older people have relatively less Type II (fast-twitch/fast-fatiguing) muscle fibres compared to young people [5]. Second, Mademli and Arampatzis [11] have suggested that older people have shorter fascicle length (they found a strong relationship between time to fatigue and muscle fascicle length) and lower ratio of active muscle volume and force production, which could be an energetic advantage. Third, metabolic mechanisms were proposed by Lanza et al. [7] who studied glycolytic ATP production in young and older people and concluded that older people who fatigued relatively slower, had improved metabolic economy (less metabolite accumulation).

Some studies have not shown significant age-related differences in fatigue [26-28], which might be explained by differences in fatigue protocols, outcomes or sample characteristics. McNeil et al. [27] found older people to be more fatigable than young during a velocity-dependent power task and suggest their contrasting findings are due to the more functionally relevant task, which employed a lighter load, and is more comparable to daily life tasks.

Explanation for the differing findings may relate to the complexity of the various processes that contribute to muscle fatigue in older people, such as decline in mitochondrial function, alterations in brain neurotransmitters, oxidative stress and inflammation [29]. The underlying mechanisms for muscle fatigue, particularly in old age are still poorly understood, and future studies are required in this area. Further research is also required to elucidate the roles played by health and fitness, the type of muscle work performed and the particular muscle groups fatigued in influencing balance control and functional task performance.

#### Effects of fatiguing different muscle groups

Knee-extensors were fatigued in six studies [19-24], of which four consistently showed consequential detrimental effects on balance and functional tasks [19-24]. This suggests that knee-extensors are important in controlling balance during quiet stance and functional tasks [21-23]. Two studies did not show a knee extensor fatigue-effect, which could be explained by the employed fatigue protocol [20,21]. The protocol of Lin et al. fatigued only one leg, which could explain why there was no effect on bilateral quiet standing [19]. The fatigue protocol of Mademli et al. involved long rest intervals (two minutes) between sets, which was not sufficiently fatiguing to find a significant reduction in muscle force [20]. Additionally, the lack of effect of knee flexor/extensor fatigue on balance recovery from a sudden release of a supported leaning posture might be explained by the fact that recovery from a leaning position predominantly involves hip flexor and/or ankle plantar-flexor muscles, more so than knee muscles [20].

Ankle plantar flexors were fatigued in four studies, of which all indicated that fatigue has a detrimental effect on standing balance, as evidenced by an increased postural sway during quiet standing [19,23,24], reduced reach [23] and reduced balance recovery following external perturbation while standing [18]. The effect of plantar-flexor fatigue on balance performance is apparent, since standing balance is regulated primarily by the ankle plantar-flexors. Furthermore, forward reaching [23] and resistance to forward postural perturbations [18] involve a large degree of plantar-flexor activity due to their antigravity action to prevent forward falling.

Lumbar extensor muscles were fatigued in two studies which both indicated that fatigue has a detrimental effect on standing balance, evidenced by increased postural sway during quiet standing [19] and increased peak COP velocity following external perturbations while standing [18]. Lumbar extensors would provide resistance to the anterior perturbations used in this study, by arresting the forward momentum of the upper body induced by the perturbation. However, the large number of outcome parameters also makes it possible that the significant effect on peak COP velocity was by chance. The study by Lin et al. [19] consistently (across multiple balance parameters) found more acute effects on standing balance from fatigue of the lumbar extensors, compared to knee-flexors and plantar-flexors. The authors suggested that back muscle fatigue might impair lumbar position sense, as well as ankle joint motion sense. This is in line with findings from Pline et al. [30] who reported that lumbar extensor fatigue may impair central processing of ankle proprioceptive signals, leading to poorer balance control. In addition, balance changes may have been more sensitive to back muscle fatigue, as this was induced bilaterally, compared to the knee and ankle muscles which were fatigued unilaterally.

Only one study investigated effect of fatigue of hip muscles on balance [24]. However, because hip abductors and extensors were fatigued in combination with muscles from other lower limb joints, it is not possible to assess the contribution that hip muscle fatigue alone had on postural sway. Further research is required to provide more insight into the role of hip muscle fatigue on balance and functional task performance.

#### Gender differences

Only one study analysed men and women separately [21]. Even though sit-to-stand power over repetitions declined more in men (29%) than women (16%) [21], however, no interaction of gender and age was reported. Hicks and McCartney [31] have suggested that women might be more fatigue resistant than men due to energy costs associated with the ratio of length-to-cross-sectional area of the muscle (women have shorter muscle length relative to cross-sectional area) or differences in the distribution of fibre type (women may have fewer Type IIB fibres). Further research is required to draw more general conclusions on gender in the effects of fatigue on balance and functional tasks.

#### Recovery from fatigue

Only one study investigated fatigue recovery rates, and indicated recovery from muscle fatigue was slower in older people compared to young [19]. However, conclusions from this study should be drawn with caution, as significant effects of muscle fatigue on quiet standing were only seen in young and not in older people. Häkkinen [8] studied recovery over 60 minutes from bilateral leg press exercise in 23 young, middle-aged and older women and found slower recovery in older people, despite a lower level of induced fatigue, relative to the younger groups. Slower recovery may put older people at risk of balance loss for a longer period. More research on the effects of recovery from fatigue for balance and functional tasks in older adults and how it relates to fall risk is required.

## Conclusions

Despite variations in study design, populations, fatigue protocols, and outcome measures findings were fairly consistent in that fatigue of the lower extremity and trunk muscles impairs balance and performance of functional tasks. This suggests that fatigue protocols could be useful in the prediction of falls; however more studies are required to decide whether muscle fatigue should be included in routine clinical fall risk assessments. More specifically, future studies should employ a common exercise to systematically examine fatigue effects of each muscle group on typical balance tasks (such as quiet standing, walking, sit to stand and responding to unexpected perturbations). This would elucidate relative effects of the different muscle groups on balance performance and assist comparisons across studies. In addition, the benefits of exercise on muscle strength and power and on recovery rate after fatigue need further investigation to indicate whether specific exercises to improve muscle fatigability should be incorporated in exercise programmes to prevent falls.

## Competing interests

The authors declare that they have no competing interests.

## Authors' contributions

All authors have read and approved the final manuscript. JLH, and MP have taken part in designing and planning of the study, data collection, data analysis and in writing the manuscript. DLS, JM, KD and SRL have taken part in designing and planning of the study, data analysis and in writing the manuscript.

## Pre-publication history

The pre-publication history for this paper can be accessed here:

http://www.biomedcentral.com/1471-2318/10/56/prepub
